# Risk communication, respiratory health risks, and air pollution forecasting in the city of Rio de Janeiro, Brazil

**DOI:** 10.36416/1806-3756/e20250168

**Published:** 2025-11-14

**Authors:** Kevin Do Hyeon Park, Kevin Cromar, Gina Gonzales, Laura Gladson, Felipe Cerbella Mandarino, Lucia Helena Barros dos Santos, Bruno Bôscaro França, Noussair Lazrak, Katherine Emma Knowland

**Affiliations:** 1. New York University Marron Institute of Urban Management, New York (NY) USA.; 2. New York University Grossman School of Medicine, Division of Environmental Medicine, New York (NY) USA.; 3. Instituto Municipal de Urbanismo Pereira Passos, Rio de Janeiro (RJ) Brasil.; 4. Secretaria Municipal do Ambiente e Clima, Rio de Janeiro (RJ) Brasil.; 5. Morgan State University, Baltimore (MD) USA.; 6. NASA Goddard Space Flight Center, Global Modeling Assimilation Office, Greenbelt (MD) USA.; 7. NASA Headquarters, Washington (DC) USA.

**Keywords:** Air pollutants/analysis, Air pollution/adverse effects, Nitrogen dioxide, Particulate matter, Developing countries, Respiratory tract diseases/epidemiology

## Abstract

**Objective:**

Although communicating air pollution risks is critical for protecting public health, particularly in low- and middle-income countries (LMICs), its effectiveness remains underexplored. This study evaluated current risk communication practices in the city of Rio de Janeiro, Brazil, by assessing the associations between short-term exposure to pollutants and respiratory-related hospital admissions; the ability of the Brazilian national índice de qualidade do ar (IQAr, air quality index) to reflect health risks; and the accuracy of pollutant forecasts in comparison with monitored concentrations.

**Methods:**

Exposure and health data for the 2014-2019 period were obtained through a research partnership with local government officials. Poisson generalized linear models were employed to determine whether IQAr values and short-term exposure to air pollutants, including nitrogen dioxide (NO_2_) and particulate matter (PM), were associated with daily hospital admissions for respiratory disease. Bias-corrected, forecasted daily concentrations of individual air pollutants from the Goddard Earth Observing System Composition Forecast Composition Forecast (GEOS-CF) model were employed to assess the performance of existing forecasting tools for use in risk communication.

**Results:**

Significant associations were consistently observed between hospital admissions for respiratory disease and short-term exposures to NO_2_ and coarse PM, with excess risks of 5.1% (95% CI: 1.3-8.9%) and 5.6% (95% CI: 1.5-9.9%), respectively, per interquartile range increases in lag day 0-1 exposures. Values of IQAr were not significantly associated with respiratory health events, likely due to their failure to capture the health risks associated with NO_2_. Bias-corrected forecasts from the GEOS-CF model showed strong correlations with observed pollutant concentrations.

**Conclusions:**

These findings indicate that adopting a health-based, multi-pollutant index, combined with improved forecasting tools, could substantially strengthen risk communication in the city of Rio de Janeiro and other LMIC settings.

## INTRODUCTION

Respiratory diseases linked to outdoor air pollution represent a significant public health burden worldwide, particularly in low- and middle-income countries (LMICs) such as Brazil. Although air quality indices are widely used to communicate pollution levels, their effectiveness in conveying short-term health risks remains underexplored. This issue is especially critical in cities that are less studied, like Rio de Janeiro, Brazil, where diverse pollution sources, unique urban geography, and socioeconomic disparities create distinct exposure patterns and health responses.^1^ ^,^ ^2^

The Rio de Janeiro *Secretaria Municipal do Ambiente e Clima* (SMAC, Municipal Secretary of the Environment and Climate) has implemented the Rio de Janeiro Air Quality Monitoring Program (MonitorAR-Rio) to inform the public of air pollution risks through dissemination of the Brazilian national *índice de qualidade do ar* (IQAr, air quality index). These efforts include informing the public of the air quality in the city through an online air quality bulletin that is updated daily with concentration values of major pollutants that the WHO has classified as having serious impacts on human health.^3^ However, the current approach using the IQAr, which is based on the pollutant with the highest calculated index value each day, has not been quantitatively assessed to determine its accuracy in reflecting population-level health risks in a way that can lead to effective decision-making by individuals regarding behavior modification. 

This study, designed and carried out in collaboration with local civil servants within the city government, addresses three key aspects of air quality risk communication: quantifying the associations between short-term exposure to pollutants and population-level health risks; evaluating the ability of the current IQAr to reflect those risks; and exploring the potential of a forecasting-based approach to enhance communication of next-day pollutant concentrations. Collectively, these findings will directly inform future decisions on how to best communicate health risks associated with outdoor air pollution in Rio de Janeiro and will provide actionable insights for air quality management in LMICs.

## METHODS

### Exposure data

For the 2014-2019 period, hourly air pollution data and IQAr values for the city of Rio de Janeiro were obtained from the SMAC. The IQAr has a theoretical range from 0 to 400, with higher values indicating higher levels of air pollution and therefore greater harm to human health. Table S1 shows the IQAr values and associated levels of health concern in accordance with the national air quality standards. Daily IQAr values are reported to the public based on the highest value associated with concentrations of the monitored pollutants.

Pollution data for this study were collected from four central monitoring stations (Centro, Irajá, São Cristóvão, and Tijuca) managed by MonitorAR-Rio. These stations were selected in consultation with local experts based on their similar geographical, meteorological, and socioeconomic characteristics to ensure consistency in exposure and health assessments. The hourly concentrations of monitored air pollutants-particulate matter with a diameter ≤ 10 µm (PM_10_), particulate matter with a diameter ≤ 2.5 µm (PM_2.5_), ground-level ozone (O_3_), carbon monoxide (CO), nitrogen dioxide (NO_2_), and sulfur dioxide (SO_2_)-were aggregated into daily exposure variables using various averaging times (see Table 1). Missing data were handled with multivariate imputation by chained equations and predictive mean matching. Hourly meteorological data, such as temperature, relative humidity, and precipitation, also provided by the SMAC (Table 1), were aggregated into daily (24-h) average variables.

**Table 1:** Descriptive statistics of air pollution and meteorological data in the city of Rio de Janeiro, Brazil. Combined data from four monitoring stations, 2014-2016.

Variable	Mean ± SD	Median (range)	IQR
NO_2_, 1-h maximum (ppb)	37.0 ± 17.3	34.0 (6.91-126.0)	20.7
O_3_, 8-h maximum (ppb)	21.7 ± 10.2	20.1 (3.74-66.3)	13.1
PM_10_, 24-h (µg/m^3^)	34.5 ± 14.1	32.0 (8.0-102.0)	17.0
PM_2.5_*, 24-h (µg/m^3^)	17.9 ± 10.7	15.0 (0.0-84.0)	12.0
SO_2_, 24-h (ppb)	1.99 ± 1.38	1.72 (0.0-13.4)	1.72
Temperature (°C)	25.3 ± 3.6	25.0 (16.0-33.7)	5.3
Daily precipitation (mm)	0.06 ± 0.26	0.0 (0.0-3.0)	0.0
Relative humidity (%)	64.6 ± 9.79	64.5 (37.3-92.5)	13.3

NO_2_: nitrogen dioxide, O_3_: ozone, PM_10_: particulate matter with a diameter ≤ 10 µm, PM_2.5_: particulate matter with a diameter ≤ 2.5 µm; SO_2_: sulfur dioxide. *PM_2.5_ data were available from only one of the four monitoring stations.

Forecasted daily concentrations of individual air pollutants from the publicly available U.S. National Aeronautics and Space Administration Goddard Earth Observing System Composition Forecast (GEOS-CF) model were employed to assess the suitability of a quantitative tool in predicting pollutant concentrations,^4^ compared with the current qualitative approach.^5^ The forecasting capabilities of the GEOS-CF were further enhanced with bias correction methods that integrate local observations through machine learning (ML).^4^ With that approach, an ML model was trained to improve upon the capabilities of the GEOS-CF model in accounting for local atmospheric trends at each monitor.^4^ ^,^ ^6^ Coefficients of determination (R^2^) were calculated for the 2018-2019 period, comparing daily monitored pollutant concentrations with forecasted concentrations from the bias-corrected GEOS-CF model.

### Health data

Daily hospital admissions for respiratory diseases during the 2014-2016 period were obtained from the data platform of the Brazilian Unified Health Care System^7^ and provided by Rio de Janeiro city officials at the Pereira Passos Institute. That period was selected in order to retain other years for independently developing and evaluating potential revisions to the current IQAr. Respiratory diseases were defined by the following ICD-10 codes: J01-J06 (acute upper respiratory infections, excluding the common cold); J18 (pneumonia, unspecified organism); J20-J22 (other acute lower respiratory infections); J30-J39 (other diseases of the upper respiratory tract); J40-J47 (chronic lower respiratory disease, including COPD and asthma); J80-J84 (other respiratory diseases principally affecting the interstitium); J86 (suppurative and necrotic conditions of the lower respiratory tract); and J90/J92-J94 (other diseases of the pleura). After patients who could not be linked back to any monitors, because of missing geographical data, had been excluded, the study sample comprised 10,431 admissions (an average of approximately 10 admissions per day).

### Statistical analysis

Time-series analyses with Poisson generalized linear models were used in order to assess the associations that the daily IQAr and individual air pollutant concentrations had with hospital admissions for respiratory disease in the city of Rio de Janeiro. Poisson generalized linear models are well-suited for analyzing count data, such as daily hospital admissions, in relation to environmental exposures. They allow the assessment of short-term associations while accounting for temporal patterns, confounding factors, lagged effects, and overdispersion, providing interpretable results, such as relative risks and excess risks, that are directly applicable to public health decisions.^8^

Optimal degrees of freedom (df) for covariates, such as temperature and seasonality, were determined by assessing model fit with the pseudo-R² and residual sum of squares. The regression model included a linear indicator for each day of the week; smooth functions of time (using natural splines) to control for seasonality and long-term trends (df = 6 per year), same-day temperature (df = 3), average temperature at lag 1-3 (df = 3), same-day relative humidity (df = 3), same-day precipitation (df = 3); and a binary indicator to control for the Rio de Janeiro 2016 Summer Olympics. Models were specified for each pollutant at individual lag days 0-5 and average lag days 0-1. Excess risks were calculated per interquartile range increase in individual pollutant concentrations and IQAr. Stability of the overall model was confirmed through sensitivity analyses of alternative df values for meteorological and seasonality variables. Two-pollutant models included a second pollutant that uses the same lag variables and indicators used in the single-pollutant model. Each possible pollutant pair was tested in the analysis. All analyses conducted were completed with RStudio, version 2022.07.1.^9^

## RESULTS

 The overall air quality of the study area in the city of Rio de Janeiro was defined by the IQAr as “good” on the vast majority of days during the study period, as “moderate” on a much smaller portion of days, and as “bad” or “very bad” on only 2% of days. More specifically, there were no days during the study period on which the NO_2_ concentrations were reported as being anything other than “good” (Table 2). 

**Table 2:** Range of the Brazilian national air quality index values (IQAr) and daily classifications for NO_2_, O_3_, PM_10_, PM_2.5_, and SO_2_ in the city of Rio de Janeiro, Brazil, 2014-2016. The Irajá monitoring station was selected as representative of the worst air quality in the region.

IQAr range	Classification	NO_2_	O_3_	PM_10_	PM_2.5_	SO_2_
		(% of days)	(% of days)	(% of days)	(% of days)	(% of days)
0-40	Good	96.0	85.0	76.0	76.0	91.0
41-80	Moderate	0.0	10.0	18.0	17.0	0.1
81-120	Unhealthy	0.0	1.7	1.4	1.6	0.0
121-200	Very unhealthy	0.0	0.3	0.0	0.1	0.0
201-400	Terrible	0.0	0.0	0.0	0.0	0.0
Missing data		4.0	3.0	4.2	5.8	8.7
Total days		1,096	1,096	1,096	1,096	1,096

NO_2_: nitrogen dioxide, O_3_: ozone, PM_10_: particulate matter with a diameter ≤ 10 µm; PM_2.5_: particulate matter with a diameter ≤ 2.5 µm; SO_2_: sulfur dioxide.

**Figure 1:**
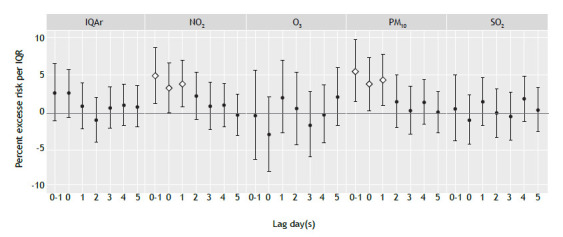
Percent excess risks of hospital admission for respiratory disease per interquartile-range increase in the Brazilian national air quality index (IQAr) and in concentrations of nitrogen dioxide (NO_2_), ozone (O_3_), particulate matter with a diameter ≤ 10 µm (PM_10_), and sulfur dioxide (SO_2_), by lag structure, in the city of Rio de Janeiro, Brazil, 2014-2016. Open diamonds indicate statistical significance (p < 0.05), and filled circles indicate statistical insignificance (p ≥ 0.05). IQAr: *índice de qualidade do ar* (air quality index).

Despite the overall air quality of the study area in the city being reported as generally healthy on most days, there were still observed increases in respiratory health risks associated with short-term exposures to NO_2_ and PM_10_. The single-pollutant models demonstrated a significant association between hospital admission for respiratory disease and air pollution exposure at lag day 0 and 1 (Figure 1). Significant, positive associations were observed for NO_2_ and PM_10_ but were not consistently found for other pollutants (O_3_ or SO_2_). Each interquartile-range (21-ppb) increase in the NO_2_ concentration was associated with an excess risk of hospital admission for respiratory disease of 5.1% (95% CI: 1.3-8.9%) at lag day 0-1; and each interquartile-range (17-µg/m^3^) increase in PM_10_ was associated with an excess risk of such admission of 5.6% (95% CI: 1.5-9.9%) at lag day 0-1. In two-pollutant models, the associations observed for NO_2_ and PM_10_ (Figures S1 and S2, respectively) were positive but attenuated when adjusted for each other. Because PM_2.5_ exposure was monitored at only one station and showed low correlation coefficients (< 0.60) with other pollutants and meteorological variables (data not shown), PM_2.5_ was excluded from the regression analysis.

Daily IQAr values reported to the public were not found to be significantly associated with population-level respiratory morbidity risks (Figure 1). During the study period, the pollutant with the highest calculated index value (Table 3), also known as the primary driver pollutant, was most commonly PM_10_ (on 611 of the 1,150 days with a primary driver pollutant) followed by O_3_ (on 353 of such days). NO_2_ was the primary driver pollutant on 14 days (Table 3), all of which were classified as having “good” air quality (Table 2) on the IQAr scale. 

**Table 3:** Number of days on which NO_2_, O_3_, PM_10_, PM_2.5_, and SO_2_ were the primary driver pollutant-which determines the Brazilian national air quality index values (IQAr)-or the secondary driver pollutant in the city of Rio de Janeiro, Brazil (from the Centro, Irajá, São Cristóvão, and Tijuca monitoring stations), 2014-2016.

Monitored pollutant	Primary driver	Secondary driver
	(days)	(days)
NO_2_	14	11
O_3_	353	243
PM_10_	611	894
PM_2.5_	146	32
SO_2_	26	23
Total days*	1,150	1,203

NO_2_: nitrogen dioxide, O_3_: ozone, PM_10_: particulate matter with a diameter ≤ 10 µm; PM_2.5_: particulate matter with a diameter ≤ 2.5 µm; SO_2_: sulfur dioxide. *The total number of days for primary and secondary drivers differs because there were some days on which two pollutants had the same calculated IQAr value.

**Figure 2:**
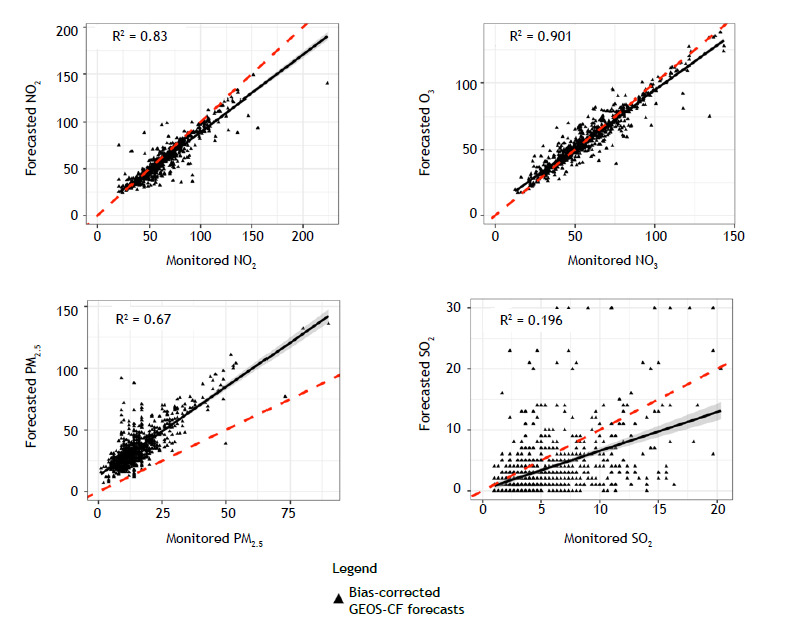
Correlation plots between monitored and forecasted levels of nitrogen dioxide (NO_2_), ozone (O_3_), particulate matter with a diameter ≤ 2.5 µm (PM_2.5_), and sulfur dioxide (SO_2_), in the city of Rio de Janeiro, Brazil, 2014-2016. Daily forecast levels of air pollutants are derived from the bias-corrected Goddard Earth Observing System Composition Forecast (GEOS-CF) model with 1-day lead time. Coefficients of determination (R^2^) are presented on each plot.

The analysis of the capability of the bias-corrected GEOS-CF model in forecasting pollutant concentrations revealed strong correlations between monitored concentrations and the bias-corrected GEOS-CF estimates (Figure 2). Those correlations were particularly strong for NO_2_ and O_3_ (R^2^ = 0.83 and 0.90, respectively). PM_10_ was excluded from the analysis because estimates of PM10 were not available in the GEOS-CF model at the time of the study.

## DISCUSSION

This study makes significant, novel contributions to the field of environmental epidemiology by evaluating the effectiveness of the IQAr in communicating short-term health risks in the city of Rio de Janeiro and proposing a more robust forecasting-based approach to improve risk communication in the city. Although traditional air quality indices have been widely used,^10^ this study is among the first to rigorously assess the inadequacies of such indices in accurately reflecting population-level health risks in an LMIC context. Although many adverse health outcomes are associated with short-term increases in ambient air pollution, respiratory morbidity has been identified as the most promising target for individual behavior modification.^11^ Therefore, respiratory morbidity was used in order to evaluate the performance of the IQAr in the present study.

The observed significant associations between short-term exposures to NO_2_ and PM_10_ and increased risks of hospital admission for respiratory disease in the city of Rio de Janeiro align closely with the findings of other studies conducted in Latin America. Previous epidemiological investigations in urban areas, such as Mexico City, Mexico and Quito, Ecuador, have similarly demonstrated that traffic-related pollutants, particularly NO_2_, are among the strongest predictors of respiratory morbidity.^8^ ^,^ ^12^ Those studies suggest that NO_2_ often serves as a marker for emissions from vehicles and other sources of combustion, which are prevalent in densely populated cities with high traffic volume in LMICs.^13^ Similarly, the association between PM and respiratory health outcomes has been consistently observed across Latin America, with PM often linked to a broad range of respiratory effects due to its ability to penetrate the respiratory tract and trigger inflammation.^14^ ^-^ ^16^ This underscores the importance of targeting reductions in NO_2_ and PM in air quality management strategies across Latin America.

Our finding that the IQAr values, driven predominantly by PM or O_3_ concentrations, fail to reflect the significant respiratory risks associated with exposure to NO_2_ is particularly striking. As previously stated, while 14 days of the three-year period had index values primarily driven by NO_2_, all of these days, however, were classified as having “good” air quality according to the IQAr classifications. This underscores the need for a health-based, multi-pollutant approach tailored to the unique urban and socioeconomic characteristics of cities like Rio de Janeiro (e.g., the strong associations between NO_2_ exposure and adverse health effects), in order to improve the effectiveness of informed behavior modification decisions to reduce exposures and health risks.

Our results highlight the need for a comprehensive risk communication framework that, at a minimum, includes the pollutants that are currently underrepresented in the IQAr system. Given that NO_2_ concentrations in Rio de Janeiro were classified as “good” under the IQAr for the entire study period, despite significant health risks, the current system may fail to adequately inform the public of potential harms.

The absence of significant associations between short-term O_3_ exposure and respiratory morbidity in our study is consistent with the findings of studies conducted in other LMIC contexts, including urban centers in other Latin America countries. Although significant associations between O_3_ and respiratory health outcomes are frequently observed in studies conducted in high-income regions, such as North America, they are less commonly reported in LMICs.^(17­­-19)^ It is noteworthy that the typical O_3_ concentrations in the city of Rio de Janeiro are substantially lower than the levels typically found in urban areas where positive associations are observed. 

The lack of significant associations observed for exposure to O_3_ in the present study does not negate the potential health risks of such exposure but rather highlights the need for further research to understand the specific conditions under which O_3_ poses a threat to respiratory health in LMIC settings. The strong influence of O_3_ on daily IQAr values represents a major opportunity to improve the health protection offered by risk communication approaches in Brazil. Although continued monitoring and analysis of O_3_ concentrations and their potential health impacts is important, ensuring that all relevant pollutants are adequately accounted for in public health frameworks is critical to refining risk communication strategies.

Our data demonstrate the potential of advanced ML-enhanced forecasting tools, such as the bias-corrected GEOS-CF model, to dramatically improve the accuracy of pollutant predictions.^20^ ^,^ ^21^ The present study provides robust evidence that real-time forecasting tools could replace the reliance on the current qualitative approach in predicting future pollutant concentrations. This could not only enhance the precision of air quality assessments but also allow more timely, actionable health messaging, thus addressing a critical limitation of current practices.

Our study has some limitations. The fact that the health outcome data used in the study did not include emergency department visits limited the statistical power of the analysis as there are typically a greater number of emergency department visits when compared with that of hospital admissions. In addition, acknowledging the diverse geographical and meteorogical characteristics of the city of Rio de Janeiro, we limited the study area to only a portion of the city, and, thus, this study may not fully reflect health risks associated with air quality in other regions.

The findings of the present study have far-reaching implications for air quality management and public health communication in LMICs. Traditional single-pollutant air quality indices have long been criticized for their inability to capture the synergistic health effects of multiple pollutants at moderate or low levels.^23^ ^-^ ^27^ This study supports a growing body of evidence advocating for the development and adoption of multi-pollutant, health-based air quality indices tailored to local contexts.^26^ ^,^ ^28^ ^-^ ^32^ By leveraging the collaborative involvement of local civil servants, this research exemplifies how partnerships between researchers and policymakers can lead to actionable solutions that improve public health outcomes.

As cities worldwide face increasing challenges from air pollution and climate variability, integrating advanced forecasting technologies with health-based indices has the potential to revolutionize air quality management and risk communication strategies. This study serves as a foundational step toward such advances, paving the way for further innovations in air pollution epidemiology and public health interventions.

Effective risk communication for outdoor air pollution requires accurate forecasting of pollutant concentrations and comprehensive consideration of the combined health risks posed by multiple pollutants. This study highlights significant gaps in the current IQAr employed in the city of Rio de Janeiro, including its inability to reflect the health risks associated with individual pollutants, specifically NO_2_, while overweighting the impact of O_3_, which was not found to be associated with increased respiratory health risks in our study. These limitations hinder the ability of the IQAr to effectively inform timely public health actions.

The results of this study provide a roadmap for specific actions that can be taken to improve risk communication in the city of Rio de Janeiro.^33^ First, our findings demonstrate the potential of advanced forecasting tools, such as the bias-corrected GEOS-CF model, to substantially improve the accuracy of pollutant predictions. High-quality forecasts for NO_2_ and PM should be immediately prioritized for use in calculating next-day index values for risk communication purposes. In addition, incorporating these tools, alongside the development of a validated, health-based multi-pollutant index, could greatly enhance the reliability of air quality information communicated to the public. There is an urgent need for improved weighting of pollutant values that better reflects the observed respiratory health risks of exposure to NO_2_ and PM. Such an advance would not only support better individual decision-making but also contribute to broader efforts to reduce the public health burden of air pollution in the city of Rio de Janeiro and similar urban settings in this and other LMICs.
